# Morphometry-based radiomics for predicting therapeutic response in patients with gliomas following radiotherapy

**DOI:** 10.3389/fonc.2023.1139902

**Published:** 2023-08-17

**Authors:** Lahanda Purage G. Sherminie, Mohan L. Jayatilake, Badra Hewavithana, Bimali S. Weerakoon, Sahan M. Vijithananda

**Affiliations:** ^1^ Department of Radiography/Radiotherapy, Faculty of Allied Health Sciences, University of Peradeniya, Peradeniya, Sri Lanka; ^2^ Department of Radiology, Faculty of Medicine, University of Peradeniya, Peradeniya, Sri Lanka

**Keywords:** radiomics, morphometry, glioma, therapeutic response, prediction model, machine learning

## Abstract

**Introduction:**

Gliomas are still considered as challenging in oncologic management despite the developments in treatment approaches. The complete elimination of a glioma might not be possible even after a treatment and assessment of therapeutic response is important to determine the future course of actions for patients with such cancers. In the recent years radiomics has emerged as a promising solution with potential applications including prediction of therapeutic response. Hence, this study was focused on investigating whether morphometry-based radiomics signature could be used to predict therapeutic response in patients with gliomas following radiotherapy.

**Methods:**

105 magnetic resonance (MR) images including segmented and non-segmented images were used to extract morphometric features and develop a morphometry-based radiomics signature. After determining the appropriate machine learning algorithm, a prediction model was developed to predict the therapeutic response eliminating the highly correlated features as well as without eliminating the highly correlated features. Then the model performance was evaluated.

**Results:**

Tumor grade had the highest contribution to develop the morphometry-based signature. Random forest provided the highest accuracy to train the prediction model derived from the morphometry-based radiomics signature. An accuracy of 86% and area under the curve (AUC) value of 0.91 were achieved for the prediction model evaluated without eliminating the highly correlated features whereas accuracy and AUC value were 84% and 0.92 respectively for the prediction model evaluated after eliminating the highly correlated features.

**Discussion:**

Nonetheless, the developed morphometry-based radiomics signature could be utilized as a noninvasive biomarker for therapeutic response in patients with gliomas following radiotherapy.

## Introduction

Glioma is the most common cancer or malignant tumor among primary brain tumors and other central nervous system tumors (1). A glioma can be life-threatening depending on the location and the rate of growth. Gliomas are graded on a scale of Grade I to IV according to the World Health Organization (2). Also, they can be categorized as slow growing (Grade I and II) and fast growing (Grade III and IV) tumors. However, usage of different glioma classifications can be seen (3–5). The aggressive forms of gliomas can result in death within few months. The complete elimination of gliomas might not be possible even after a treatment due to their complex and infiltrative nature. Therefore, assessment of therapeutic response is important to determine the efficacy of the given treatment and for future decision-making. Thus, the predictive analytics related to clinical outcomes such as therapeutic response has become popular in the clinical setting. Even though there are predictive biomarkers such as isocitrate dehydrogenase 1 (IDH) and 1p/19q co-deletion used in gliomas considering their molecular profiling (6–8) the use of molecular biomarkers has practical barriers for the wide application due to the cost of testing as well as limited resources. In contrast, an imaging biomarker such as radiomics is less expensive and non-invasive. Hence, application of radiomics is more favorable compared to molecular biomarkers. Nevertheless, radiomics itself is not a mature field (9). Especially there were limited number of studies with respect to the application of radiomics studies to assess the therapeutic response in patients with gliomas after receiving radiotherapy. Moreover, most of the studies had focused on non-morphometric features compared to morphometric features. Since only morphometric features can reflect the geometric aspects of a tumor there is a need to further investigate the usefulness of morphometry-based radiomics features for making predictions in disciplines like radiotherapy. Hence, this study was aimed at developing a prediction model using a morphometry-based radiomics signature to predict therapeutic response for patients with gliomas following radiotherapy and exploring whether the model performance was affected by the highly correlated features.

## Results

The clinical information related to therapeutic response was available for 105 patients and that information was missing for 5 patients. Thus, the patients without relevant information and irrelevant information were excluded from this study. In this dataset gliomas were classified as astrocytoma, oligodendroglioma, mixed glioma and glioblastoma multiforme (GBM). Also, they were graded on a scale of Grade II to IV. According to the clinical data the presence and absence of tumor after treatment indicated 81% and 19% respectively. Among the selected patients 54% of them were males and 46% of them were females. **Table 1** shows further demographic and clinical information related to this study sample.

**Table 1:** Characteristics of patients in this study sample.

Characteristics	Number of patients (%), n = 105
Age (years) Mean ± standard deviation Range	52 ± 15 18 – 76
Gender Male Female	57 (54.29) 48 (45.71)
Race White Black or African American Asian Not available	93 (88.57) 8 (7.62) 2 (1.90) 2 (1.90)
Vital status Alive Dead	43 (40.95) 62 (59.05)
Glioma classification • Astrocytoma - Supratentorial, frontal lobe - Supratentorial, temporal lobe - Supratentorial, parietal lobe • Oligodendroglioma - Supratentorial, frontal lobe - Supratentorial, temporal lobe - Supratentorial, parietal lobe • Mixed glioma - Supratentorial, frontal lobe - Supratentorial, temporal lobe - Supratentorial, parietal lobe • Glioblastoma multiforme	15 (14.29) 7 (6.67) 5 (4.76) 3 (2.86) 14 (13.33) 10 (9.52) 3 (2.86) 1 (0.95) 14 (13.33) 6 (5.71) 7 (6.67) 1 (0.95) 62 (59.05)
Glioma grade II III IV	14 (13.33) 29 (27.62) 62 (59.05)
Median survival Grade II Grade III Grade IV	117.4 months 3.9 months 15.6 months
Type of radiation External beam radiotherapy Other (i.e., implants, combination of methods)	102 (97.14%) 3 (2.86%)
Fractionation schedules 63 Gy in 35# 61.2 Gy in 34# 61 Gy in 33# 61 Gy in 34# 60 Gy in 30# 60 Gy in 50# 60 Gy in 60# 60 Gy in 33# 60 Gy in 23# 59.4 Gy in 33# 59.4 Gy in 30# 57 Gy in 30# 54 Gy 27# 54 Gy 30# 50.4 Gy 28# 50.4 Gy 30# 18 Gy in 1# Not available	1 (0.95) 1 (0.95) 1 (0.95) 1 (0.95) 53 (50.48) 1 (0.95) 2 (1.90) 1 (0.95) 1 (0.95) 11 (10.48) 3 (2.86) 1 (0.95) 3 (2.86) 5 (4.76) 1 (0.95) 1 (0.95) 1 (0.95) 17 (16.19)

**Figure 1:**
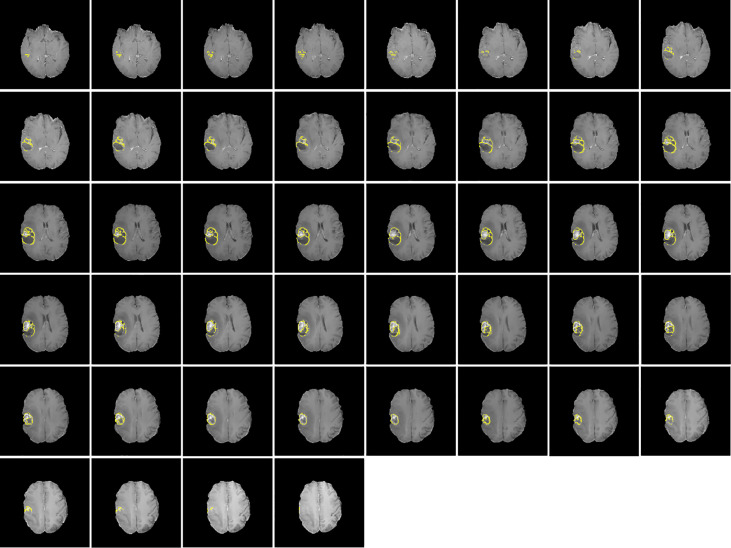
Contoured ROIs (yellow) showing GBM on contrast enhanced T1-weighted MR images. Adapted from CBICA Image Processing Portal; https://ipp.cbica.upenn.edu/. A web accessible platform for imaging analytics; Center for Biomedical Image Computing and Analytics, University of Pennsylvania.

**Figure 2:**
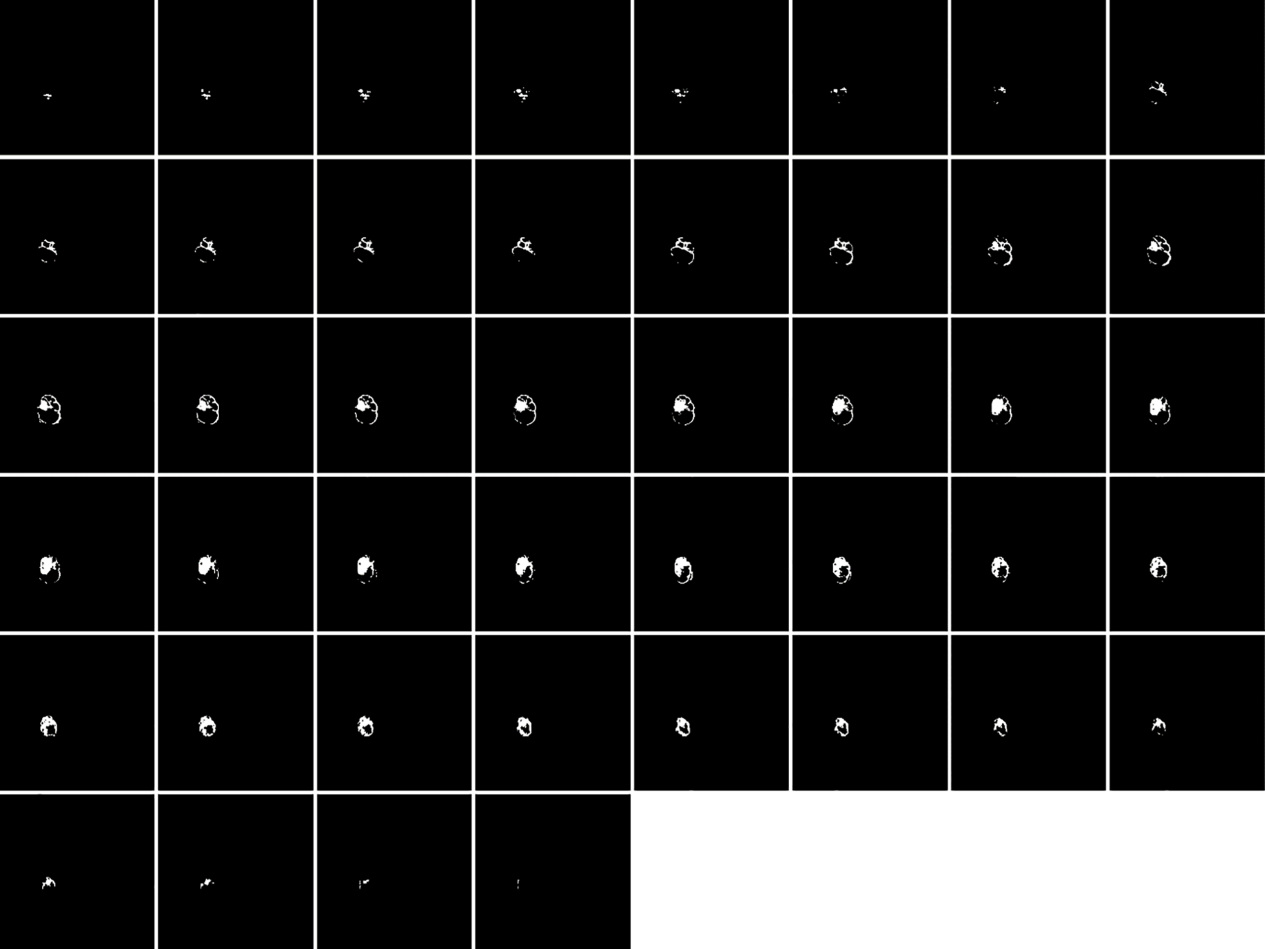
Segmented ROIs corresponding to the contoured ROIs shown in Figure 1. Adapted from CBICA Image Processing Portal; https://ipp.cbica.upenn.edu/. A web accessible platform for imaging analytics; Center for Biomedical Image Computing and Analytics, University of Pennsylvania.

**Figure 3:**
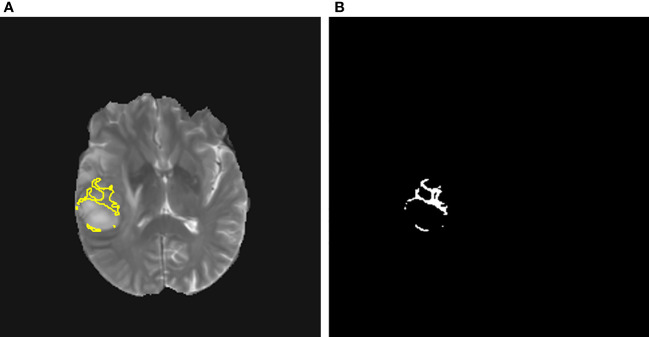
**(A)** A contoured ROI (yellow) and **(B)** corresponding segmented ROI are shown (Magnification ×15; **(A)** relative to a slice in **Figure 1** and **(B)** relative to a slice in **Figure 2**).

**Figure 4:**
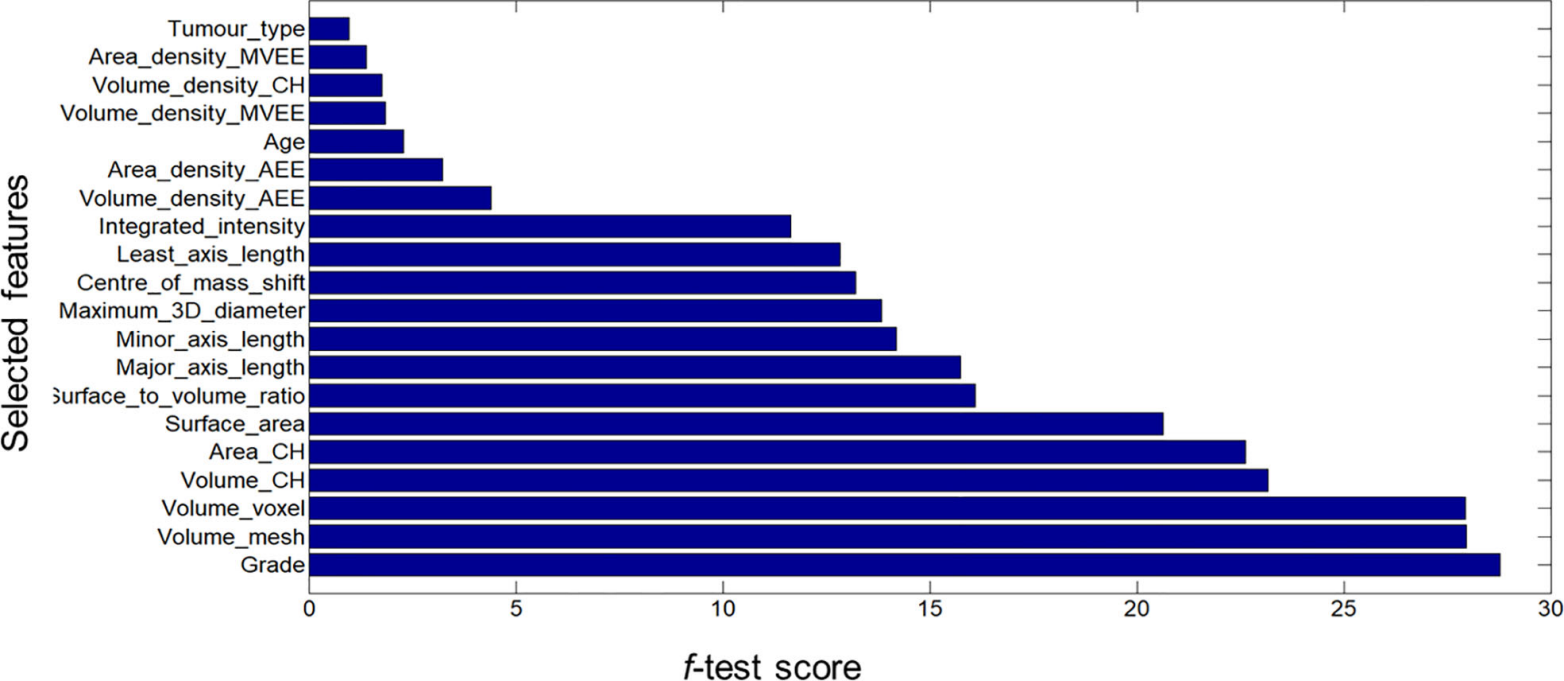
Bar graph showing the role of individual features that contributed to the developed radiomics signature.

**Figures 1**, **2** display an example of the contoured tumor ROIs (10) on a series of MR image slices in Neuroimaging Informatics Technology Initiative (NIfTI) format and the corresponding segmented ROIs obtained from a patient with GBM. **Figure 3** displays resized images with a contoured ROI and corresponding segmented ROI taken from **Figures 1**, **2**. Similar examples are shown in **Supplementary Figure 1–6** Twenty features having the highest impact on predicting the therapeutic response were selected as most predictive features according to the scores obtained from ANOVA *f*-test. The contribution of those individual features for the development of radiomics signature is illustrated in the **Figure 4** and tumor grade had the highest impact tumor type for developing the radiomics signature.

Based on these scores a Rad-score was calculated as given below.

Rad-score = Grade*28.7848 + Volume_mesh*27.9530 + Volume_voxel *27.9388 + Volume_CH*23.1763 + Area_CH*22.6319 + Surface_area*20.6406 + Surface_to_volume_ratio*16.1088 + Major_axis_length*15.7395 + Minor_axis_length*14.1963 + Maximum_distance*13.8473 + Centre_of_mass_shift*13.2133 + Least_axis_length*12.8249 + Integrated_intensity*11.6341 + Volume_density_AEE*4.3956 + Area_density_AEE*3.2322 + Age*2.2917 + Volume_density_MVEE*1.8574 + Volume_density*CH_1.7642 + Area_density_MVEE*1.3962 + Tumor_type*0.9832

The selected machine learning algorithm was random forest as it indicated the highest classification accuracy (81.59%) according to **Table 2**. A significant difference in accuracy was demonstrated between Gaussian naïve Bayes and all the other algorithms. i.e., logistic regression and Gaussian naïve Bayes (p-value = 0.006), linear discriminant analysis and Gaussian naïve Bayes (p-value = 0.001), k-nearest neighbors classifier and Gaussian naïve Bayes (p-value = 0.012), classification and regression tree and Gaussian naïve Bayes (p-value = 0.032), support vector machine and Gaussian naïve Bayes (p-value = 0.001), random forest and Gaussian naïve Bayes (p-value = 0.003).

**Table 2:** Performance of tested algorithms using cross validation.

Algorithm	Accuracy (%)	Standard deviation (SD)
Logistic regression	77.27	0.11
Linear discriminant analysis	81.52	0.10
k-nearest neighbors classifier	76.44	0.10
Classification and regression tree	77.35	0.10
Gaussian naïve Bayes	67.20	0.08
Support vector machine	78.11	0.07
Random forest	81.59	0.09

**Table 3** presents the performance evaluation metrics of the trained model before optimizing the hyperparameters whereas **Table 4** presents the performance evaluation metrics of the trained model after optimizing the hyperparameters. The classification reports obtained after both random search and grid search were similar (**Table 4**). The initial accuracy was 82% according to **Table 3** and an accuracy of 86% was achieved after optimizing hyperparameters. Hence, the finally constructed model was able to predict the absence or presence of tumor after radiotherapy with 86% accuracy. The performance metrics in terms of precision, recall, f1-score for the prediction of absence of tumor yielded 91%, 81% and 86% respectively. The precision, recall, f1-score for the prediction of presence of tumor yielded 82%, 92% and 87% respectively (**Table 4**).

**Table 3:** Classification report for the model performance prior to hyperparameter tuning.

	Precision (%)	Recall (%)	F1-score (%)	Support
Absence of tumor	87	77	82	26
Presence of tumor	79	88	83	25
Accuracy			82	51
Macro average	83	82	82	51
Weighted average	83	82	82	51

**Table 4:** Classification report for the model performance after hyperparameter tuning.

	Precision (%)	Recall (%)	F1-score (%)	Support
Absence of tumor	91	81	86	26
Presence of tumor	82	92	87	25
Accuracy			86	51
Macro average	87	86	86	51
Weighted average	87	86	86	51

**Figure 5:**
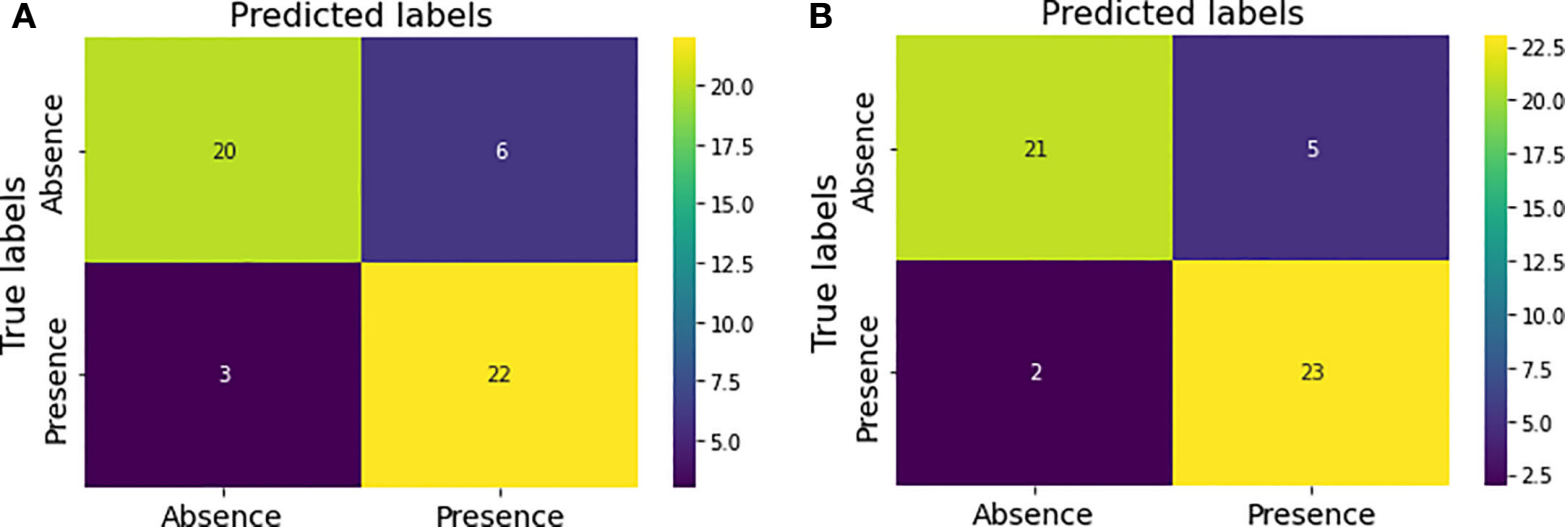
Confusion matrices for the model performance **(A)** prior to hyperparameter tuning **(B)** after hyperparameter tuning.

**Figure 5A** shows the confusion matrix prior to hyperparameter tuning. It shows that the initial model was able to predict 20 responses correctly as “absence of tumor” and 22 responses correctly as “presence of tumor” with 3 false negatives and 6 false positives. Based on this confusion matrix following performance metrics were calculated using the equations (3) (4) (5) and (6). The achieved accuracy was 82%. Precision, recall and f1-score were calculated separately for the two classes (i.e., absence of tumor and presence of tumor). For the absence of tumor precision was 87%, recall was 77% and f1-score was 82%. Likewise, precision, recall and f1-score for presence of tumor were 79%, 88% and 83%, respectively. Using the confusion matrix shown in **Figure 5B** these performance metrics were calculated for the model after hyperparameter tuning as well. The confusion matrices obtained after both random search and grid search were similar (**Figure 5B**). Accordingly final model achieved an accuracy of 86%. The precision, recall and f1-score were 91%, 81% and 86%, respectively for the absence of tumor and 82%, 92% and 87%, respectively for the presence of tumor.

The constructed model was able to predict the therapeutic response with an AUC value of 0.92 according these ROC curves. When the model was evaluated after grid search AUC value was 0.91.

**Figure 6:**
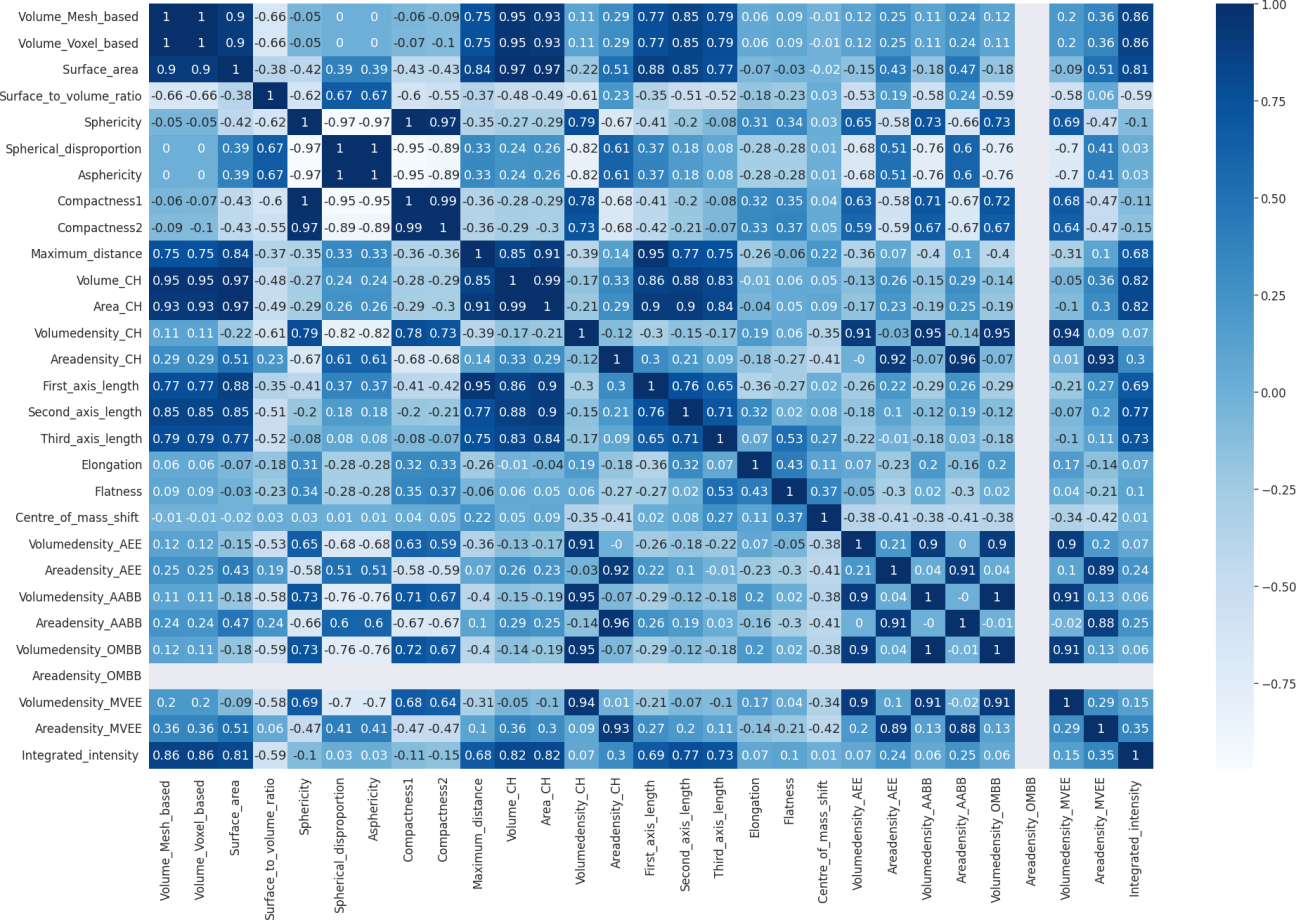
Correlation matrix.

During the process of model development after elimination of highly correlated features, the correlation matrix shown in **Figure 6** was obtained. According to it voxel-based volume, spherical disproportion, asphericity, compactness 1, compactness 2, convex hull volume, convex hull area, volume density based on AABB, area density based on AABB and volume density based on OMBB were identified as highly correlated features. After removing these features, a morphometry-based radiomics signature was developed with the sixteen features using the highest ANOVA *f-*test scores. Therefore, the new Rad-score could be given as:

Rad-score = Grade*28.7848 + Volume_mesh*27.9530 + Surface_area*20.6406 + Surface_to_volume_ratio*16.1083 + Major_axis_length*15.7395 + Minor_axis_length*14.1963 + Maximum_3D_diameter*13.8473 + Centre_of_mass_shift*13.2133 + Least_axis_length*12.8249 + Integrated_intensity*11.6341 + Volume_density_AEE*4.3956 + Area_density_AEE*3.2322 + Age*2.2917 + Volume_density_MVEE*1.8574 + Volume_density_CH*1.7642 + Area_density_MVEE*1.3962

Random forest exhibited higher accuracy (i.e., 83.26%) compared to other algorithms during ten-fold cross validation (**Supplementary Data**). Therefore, it was identified as the most appropriate algorithm to train the prediction model in this approach as well (**Table 5**). However, there was no significant difference between any of the tested algorithms.

**Table 5:** Performance of tested algorithms using cross validation to develop new model.

Algorithm	Accuracy (%)	Standard deviation (SD)
Logistic regression	78.94	0.11
Linear discriminant analysis	81.44	0.12
k-nearest neighbors classifier	78.11	0.11
Classification and regression tree	78.18	0.10
Gaussian naïve Bayes	74.70	0.11
Support vector machine	79.85	0.09
Random forest	83.26	0.07

In this approach the developed prediction model achieved an accuracy of 82% with the random forest before optimizing hyperparameters (**Table 6**). Precision, recall and f-score for absence of tumor after treatment were 84%, 81% and 82% respectively and for presence of tumor after treatment were 81%, 84% and 82% respectively. The achieved accuracy after hyperparameter tuning using grid search was 84% with precision, and f1-score for absence of tumor after treatment indicating 91%, 77% and 83% respectively and for presence of tumor after treatment indicating 79%, 92% and 85% respectively (**Table 7**).

**Table 6:** Classification report for the new model performance prior to hyperparameter tuning.

	Precision (%)	Recall (%)	F1-score (%)	Support
Absence of tumor	84	81	82	26
Presence of tumor	81	84	82	25
Accuracy			82	51
Macro average	82	82	82	51
Weighted average	82	82	82	51

**Table 7:** Classification report for the new model performance after hyperparameter tuning.

	Precision (%)	Recall (%)	F1-score (%)	Support
Absence of tumor	91	77	83	26
Presence of tumor	79	92	85	25
Accuracy			84	51
Macro average	85	84	84	51
Weighted average	85	84	84	51

**Figure 7:**
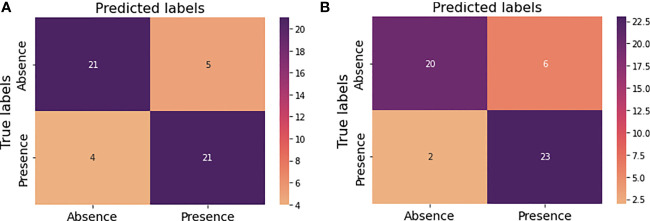
Confusion matrices for the new model performance **(A)** prior to hyperparameter tuning **(B)** after hyperparameter tuning.

**Figures 7A, B** illustrate the confusion matrices before and after hyperparameter tuning for this new prediction model. As before same performance metrics could be calculated from these confusion matrices.

This model was able to predict the therapeutic response with an AUC-ROC of 0.92 after grid search.

## Discussion

This study focused on developing a morphometry-based radiomics signature for the prediction of therapeutic response in patients with glioma after receiving radiotherapy. A prediction model was built using the most predictive features without removing the highly correlated features as well as removing the highly correlated features. The model built after eliminating the highly correlated features yielded a lesser accuracy (84%) compared to the model built without removing correlated features (86%). Therefore, elimination of the highly correlated features had not improved the accuracy. However, AUC value for the model built after eliminating highly correlated features was slightly higher (0.92) than the AUC value obtained without eliminating the highly correlated features (0.91). In addition, random search and grid search yielded similar values for the accuracy, precision, recall and f1-score but slightly different values for AUC when evaluated for the model built without eliminating highly correlated features.

In addition to the morphometric features age, gender, tumor type and grade also exhibited prediction ability according to this study. Especially tumor grade has shown the highest contribution to the developed signature. Previous studies had also utilized the demographic or clinical features to construct models aiming to improve the outcome prediction. For example, the model constructed by Patel et al. also included both clinical features such as age and radiomics features to evaluate the prediction of therapeutic response in patients with GBM following radiotherapy. Their sample was also inclusive of TCIA data. Further, they had identified sphericity and elongation as important morphometric features to build the model whereas this study identified mesh-based volume, surface area etc. are more important compared to those two features. While this study used ANOVA *f*-test for feature selection and random forest to train the model they had used random forest for feature selection and Naïve Bayes to train the model. Besides their AUC value of 0.8 and accuracy of 74% were lesser than the AUC value and accuracy obtained in this study (11).

Pan et al. also developed a radiomics signature to predict the response of 152 patients having GBM treated with radiotherapy and only one morphometric feature, i.e., minor axis length was included as a predictive feature. In contrast this study included 105 patients with glioma following radiotherapy and several morphometric features were selected to build the radiomics signature. They too had included data from TCIA. Unlike this study they had used Boruta for the feature selection. They had used several machine algorithms including random forest whereas the random forest was determined as the most appropriate algorithm to train the model in this study. In addition to the grid search which they had used, random search was also used for hyperparameter optimization in this study. However, they had achieved an AUC of 0.98 and an accuracy of 94% both of which were higher than the values obtained in this study (12). Another study had evaluated therapeutic response after chemoradiotherapy in GBM patients who were selected from TCIA as well. Similar to our study they too had used ANOVA for feature selection. Yet, they had selected linear discriminant analysis to develop the model. However, their accuracy (82.3%) is lesser than the accuracy achieved in this study but the AUC value is slightly higher than that of this study (92%). Besides, they had constructed their model based on non-morphometric features (13). Zhang et al. had developed four models to predict therapeutic response for patients with postoperative residual gliomas treated with chemoradiotherapy but none of them had identified any morphometric feature as a predictive feature. Nevertheless, they had built the prediction models using multiple logistic regression and only one model had better AUC with low accuracies for all four models (14).

Thus, the results obtained in our study are comparable to previously conducted studies in certain areas and differ in some areas. In addition, there were a limited number of studies specifically related to patients with gliomas following radiotherapy and they were also focusing mostly on non-morphometric features such as texture, wavelet, etc. According to the findings in this study morphometric features showed a higher contribution for the developed radiomics signature indicating their predictiveness of therapeutic response following radiotherapy. The prediction accuracy of 86% and AUC value of 0.91 concludes that morphometry-based signature could be utilized as a noninvasive biomarker for therapeutic response in patients with glioma. According to this study the elimination of highly correlated morphometric features did not improve the accuracy of the prediction model.

Not only high-quality images (i.e., images without artifacts and distortions) clinical information such as therapeutic response is also required for making predictions and finding such databases with reliable data was practically difficult. Furthermore, radiotherapy is an integral component in the treatment of gliomas but there are other systemic therapies such as chemotherapy that might be used as treatment options. If such treatment was used, that also affect the therapeutic response. Due to the unavailability of treatment specific information the confounding effects of such treatment were not considered in this study. Further, the application of machine learning algorithms, which is the current trend in predictive modelling, needs large sample size for training and evaluating the model. Having access to large sample sizes is practically difficult and it was a major limitation in this study as well.

Like radiomics deep learning, a subfield of machine learning, is rapidly gaining worldwide popularity and it is successfully applied in various areas. Unlike machine learning it is more efficient and has the capability to produce extremely high-level data representations. However, these advantages of deep learning highly depend on massive amounts of data (15). Therefore, machine learning is preferable for this study since our dataset is not that large. Furthermore, computational demand is comparatively high for deep learning. Thus, the performance is limited by the available computing power (16). In that perspective also machine learning was more appropriate for our task. Even though deep learning makes it less suitable for our study considering the above facts future direction of predicting therapeutic response should focus on building a deep learning model with large dataset. Nevertheless, establishment of large-scale datasets is mandatory for building prediction models based on either machine learning or deep learning techniques.

Moreover, the external validation is important to determine the model’s reproducibility and generalizability. Yet, it was not possible to find the appropriate data which was suitable for this study. Therefore, it is another limitation in this type of study. On the other hand, prediction models are never truly validated as pointed out by Calster et al. (17).

In addition, consideration of the clinical utility of radiomics models are also important. Up to date a large number of studies were done but implementation in clinical context is still challenging. Still radiomics is not clinically implemented since there are limitations for integrating radiomics in radiotherapy practice (18). There are advantages of using morphometric features compared to other categories of radiomics features. For example, morphometric features are insensitive to normalization (19) as well as to pixel space resampling or interpolation (20). Also, they were less affected by the noise which is favorable for their utilization in radiation oncology (21).

The robustness and repeatability of these features are also important for achieving the optimum benefit in clinical applications and morphometric features had exhibited highest repeatability and robustness (22–24). However, there are factors affecting their repeatability and robustness. For example, the image sequence or image contrast may impact the robustness and repeatability of a morphometric feature for a particular study (19, 25). In addition to the type of image (26, 27), image acquisition parameters (28) and software platform (29) could also affect the reliability of these features. Moreover, the robustness of extracted morphometric features may vary depending on the method of segmentation (30–33). Delineation or the contouring of ROI is mostly done manually and that is a time-consuming procedure. Both manual and semi-automatic segmentation are subject to interobserver variability as well as intra-observer variability affecting the reproducibility and reliability of radiomics analysis (34–37). Lack of standardization and harmonization methods is also a problem in obtaining reliable results (38).

Therefore, it is necessary to take the above factors into consideration when incorporating morphometric features into a radiomics model which would be clinically implementable and acceptable.

## Methods

110 patients with pathologically confirmed gliomas following radiotherapy were retrospectively evaluated. All the patients had magnetic resonance imaging (MRI) scans prior to treatment and had received radiotherapy for the primary tumor site. All the images were obtained as multimodal scans (i.e., T1-weighted, Gd-enhanced T1-weighted, T2-weighted, T2-weighted FLAIR) (39). Therapeutic response was obtained from clinical data during treatment follow-up and the response after radiotherapy to primary tumor site was considered as the therapeutic response for this study excluding response after radiotherapy to recurrent tumor or reirradiation.

This study used segmented as well as non-segmented deidentified images and clinical data obtained from Brain Tumor Segmentation (BraTS) datasets (10), The Cancer Imaging Archive (TCIA) and Genomic Data Commons (GDC) Data Portal (40) in compliance with their data usage policies and restrictions (39, 41–45). Segmented images referred to as already segmented glioma regions of interest (ROIs) obtained from the BraTS dataset. Those segmentations had been done manually and were approved by expert board-certified neuroradiologists. The non-segmented images are the images obtained without ROI delineation and then ROIs were segmented manually. However, all the images were reviewed by an experienced board-certified radiologist after segmentation. Then necessary changes were incorporated prior to feature extraction.

The image processing and feature extraction were performed for all the patients using MATLAB 2014a. Normalization with respect to signal intensities and voxel sizes were not considered in this study prior to feature extraction as they do not have a huge impact on morphometric features (19, 46). According to the previous studies there was no uniformity in selecting the morphometric features or morphometric features being identified as predictive features. Therefore, this study included 29 morphometric features according to the image biomarker standardization initiative (IBSI) (47) considering their potential to yield different outcomes.

**Figure 8:**
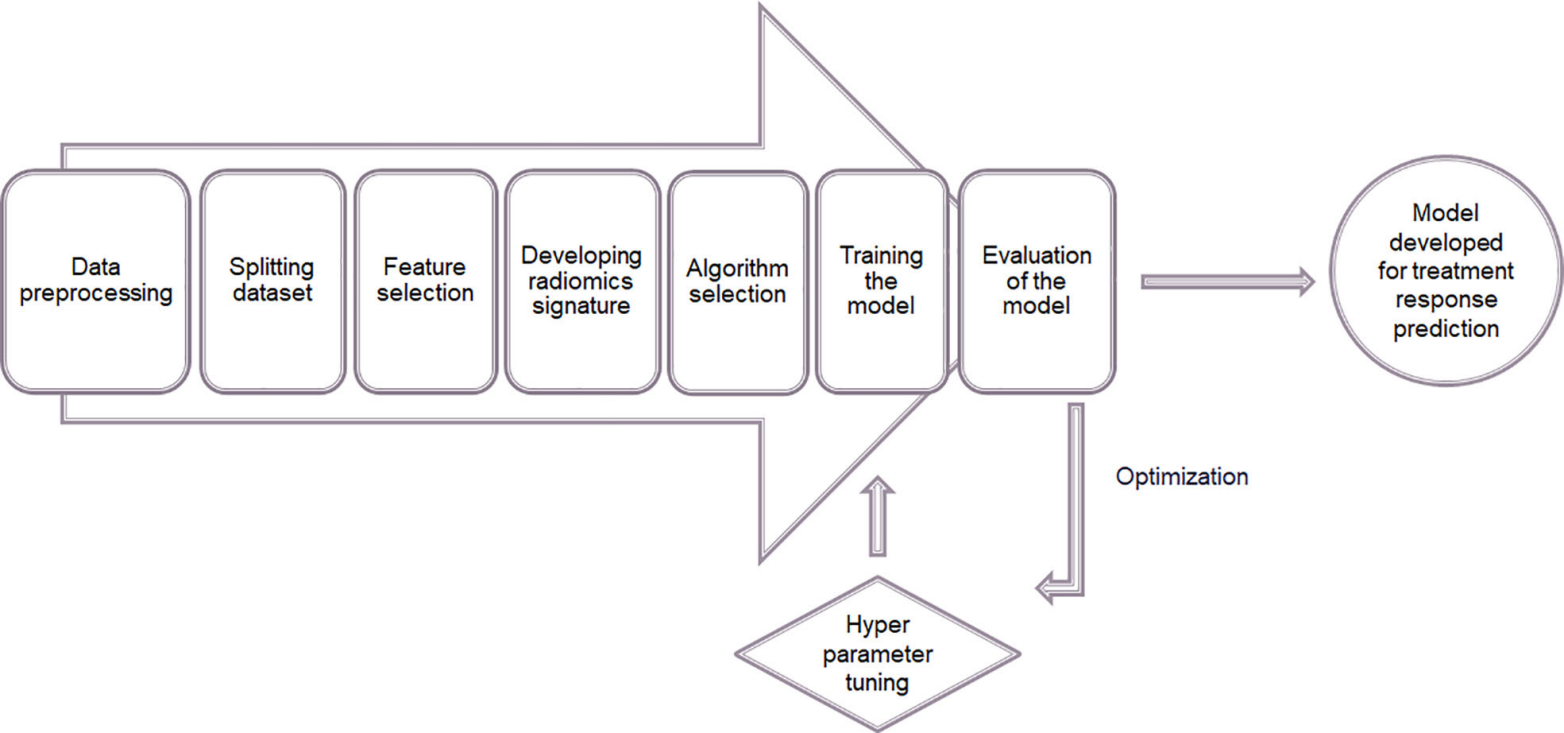
Process of constructing a model for predicting therapeutic response using supervised machine learning.

After feature extraction supervised learning method was applied to build a model for predicting therapeutic response following radiotherapy. The feature analysis including the model construction was done using Python 3.7. The steps of constructing the model are illustrated in the **Figure 8**.

During the data preprocessing stage data was cleaned or tidied first in order to facilitate analysis. The unwanted and irrelevant observations as well as duplicate observations were removed, missing values were identified with a heatmap and removed accordingly to clean the data. Then the variables such as gender and therapeutic response were labeled. For example, therapeutic response had been evaluated in terms of absence and presence of tumor following radiotherapy. Such information was collected using the standard forms and documents (48). Since that could be treated as a binary classification, they were labeled using two tags. In addition, it was ensured that the dataset was structured in a manner so that each column, row and cell represented a variable, an observation and a single value respectively. The unequal distribution between the two classes (i.e. presence of tumor and absence of tumor following radiotherapy) can lead to over classification and the model will be biased towards the majority class. Therefore, synthetic minority oversampling technique (SMOTE) was used as a measure of solving class imbalance prior to model construction (49). Then features of the entire dataset were normalized to ensure the consistency. Here, the normalization refers to having zero mean value with unit variance for all the feature values. With normalization the values were rescaled and the significance of any outliers that might present due to the broad range of feature variabilities were reduced. Following equation gives the normalized feature value x


(1) $$\begin{array}{c} x\;=\frac{X-\min\left(X\right)}{\max\left(X\right)-\min\left(X\right)} \end{array}$$

where X is the original value.

After normalization the dataset was divided into two sets as training (70%) and test (30%) datasets. The train-test split was stratified to preserve the same proportions of data in each class as observed in the original dataset. In addition to the morphometry-based features age, gender, tumor type and grade were also included to find out their potential for predicting therapeutic response. Then the most predictive twenty features to forecast the therapeutic response were selected from the training dataset using Analysis of Variance (ANOVA) *f*-test. ANOVA *f*-test was used to compare the variances between the two classes and within the classes for each feature and determine whether there was a significant difference between the two classes for that particular feature. The ratio between these two variances were given as the *f*-test score. Therefore, *f*-test score can be give as:


(2) $$\begin{array}{c} f-test\;score\;=\frac{Variance\;between\;the\;classes\;\left(Mean\;Square\;for\;Classes\right)}{Variance\;within\;the\;classes\;\left(Mean\;Square\;for\;Error\right)} \end{array}$$

The *f*-test score reflects how much it impacts the therapeutic response. Hence, a radiomics signature was developed based on *f*-test scores of the selected features. After that, the most appropriate machine learning algorithm to predict the therapeutic response was selected using ten-fold cross validation to validate the efficiency of the model that was going to be constructed. Since the evaluation of therapeutic response involved two output classes only classification machine learning algorithms such as logistic regression, linear discriminant analysis, k-nearest neighbor, classification and regression trees, Gaussian naïve Bayes, support vector machine and random forest were used to determine the most suitable algorithm in this study.

The average accuracy was calculated for all the above mentioned algorithms. The algorithm with the highest accuracy was chosen to train the prediction model. In addition, the two-sample *p*-value test (one-tailed) with 95% confidence level was performed to determine whether there was a significant difference between the accuracies of the tested algorithms. Using the most appropriate machine learning algorithm and the selected normalized features the prediction model was trained for the developed radiomics signature. Once the model was constructed its performance was evaluated in terms of accuracy, precision, recall and f1-score using the test dataset. Accuracy is the ratio of the number of correct predictions to the total number of predictions and it can be given as:


(3) $$\begin{array}{c} Accuracy=\;\frac{TP+TN}{TP+FP+TN+FN} \end{array}$$

where TP is true positive, TN is true negative, FP is false positive and FN is false negative.

Precision is the ratio of the correct positive predictions to the total positive predictions and it can be given as:


(4) $$\begin{array}{c} Precision=\;\frac{TP}{TP+FP} \end{array}$$

Recall or sensitivity is the ratio of correct positive predictions to the actual positive predictions and it can be given as:


(5) $$\begin{array}{c} Recall=\;\frac{TP}{TP+FN} \end{array}$$

f1 - score is the weighted average of precision and recall. It can be given as:


(6) $$\begin{array}{c} F1-score=\;2*\frac{Precision*Recall}{Precision+Recall} \end{array}$$

Then the hyperparameters i.e., the parameters that reflect the structure of the model, were tuned considering the obtained results as a baseline in performance with default hyperparameter settings. The hyperparameter tuning is the process for determining the appropriate combination of parameters which maximizes the model performance. During this optimization procedure the model configuration that resulted in the optimal performance in terms of maximizing accuracy or minimizing error was found out. In this study random search and grid search were used to optimize the hyperparameters. While random search finds out the optimal hyperparameters from random points in the parameter grid defined by the bounded domain of hyperparameter values, grid search finds out the optimal hyperparameters from every point in the parameter grid. After optimizing the hyperparameters the model performance was evaluated again using the area under curve-receiver operating characteristic (AUC-ROC) curve in addition to the previously mentioned performance evaluation metrics.

Next, the above mentioned process was repeated to develop a prediction model after removing highly correlated features. To eliminate the highly correlated features a correlation matrix was computed by calculating correlation coefficients for each morphometric feature during data preprocessing and the dependence of one morphometric feature on another was identified. Then highly correlated features as indicated by the correlation coefficients greater than 0.95 were removed. Then the rest of the steps were repeated as before.

## Data availability statement

Publicly available datasets were analyzed in this study. This data can be found here: https://www.cancerimagingarchive.net/collections/.

## Author contributions

MJ designed the study. LS collected the data and draft the manuscript. MJ, LS and SV performed the analysis. MJ, BH, and BW critically reviewed the manuscript. All authors contributed to the article and approved the submitted version.
