# Supplementary material for: Morphometry-based radiomics for predicting therapeutic response in patients with gliomas following radiotherapy
Source: Front Oncol. 2023 Aug 17;13:1139902. doi: 10.3389/fonc.2023.1139902 (PMC10470056; doi:10.3389/fonc.2023.1139902)
Supplement: Supplementary file 1 [file DataSheet_1.docx]

Supplementary Material

# Supplementary Table 1. Morphometric features used in this study

| Morphometric feature | Definition of feature |
| --- | --- |
| Mesh-based volume  (*Volume_mesh*) | Volume calculated by summation of each tetrahedron that forms the polygon mesh and is defined as  $V= \left\vert\sum_{k=1}^{N_{f}} V_{k} \right\vert$  where $V_{k}$ is the signed volume of tetrahedron $k$ and $N_{f}$ is the number of faces forming the surface mesh of the ROI |
| Voxel-based volume  (*Volume_voxel*) | Volume calculated by counting the number of voxels in the ROI and is defined as  $V_{voxel}= \sum_{k=1}^{N_{v}} V_{k}$  where $V_{k}$ is the volume of voxel $k$ and $N_{v}$ is the number of voxels |
| Surface area | $A=\sum_{k=1}^{N_{f}} A_{k}$  where $A_{k}$ is the area of face $k$ |
| Surface-to-volume ratio | $\frac{A}{V}$ |
| Sphericity | $\frac{\left( 36\pi V^{2} \right)^{\frac{1}{3}}}{A}$ |
| Spherical disproportion | $\frac{A}{\left( 36\pi V^{2} \right)^{\frac{1}{3}}}$ |
| Asphericity | $\left( \frac{1}{36\pi}\frac{A^{3}}{V^{2}} \right)^{\frac{1}{3}}-1$ |
| Compactness 1 | $\frac{V}{\pi^{\frac{1}{2}}A^{\frac{3}{2}}}$ |
| Compactness 2 | $36\pi\frac{V^{2}}{A^{3}}$ |
| Maximum 3D diameter | $\max\left( \left\Vert\vec{X}_{conv,k_{1}}-\vec{X}_{conv,k_{2}} \right\Vert_{2} \right), k_{1}=1, . .,N k_{2}=1, . . ,N$  where $X_{conv}$ is the set of points / vertices on the surface of the convex hull |
| Convex hull-based volume  (*Volume_CH*) | $V_{convex}=\left\vert\sum_{k=1}^{N_{f}} V_{k} \right\vert$  where $V_{k}$ is the signed volume of tetrahedron $k$ and $N_{f}$ is the number of faces forming the convex hull |
| Convex hull-based area  (*Area_CH*) | $A_{convex}=\sum_{k=1}^{N_{f}} A_{k}$  where $A_{k}$ is the area of face $k$ of convex hull |
| Convex hull-based volume density  (*Volume_density_CH*) | $\frac{V}{V_{convex}}$ |
| Convex hull-based area density | $\frac{A}{A_{convex}}$ |
| Major axis length | $4\sqrt{\lambda_{major}}$  where $\lambda_{major}$ is the largest eigenvalue obtained by principal component analysis (PCA) |
| Minor axis length | $4\sqrt{\lambda_{minor}}$  where $\lambda_{minor}$ is the second largest eigenvalue obtained by PCA |
| Least axis length | $4\sqrt{\lambda_{least}}$  where $\lambda_{least}$ is the smallest eigenvalue obtained by PCA |
| Elongation | $\sqrt{\frac{\lambda_{minor}}{\lambda_{major}}}$ |
| Flatness | $\sqrt{\frac{\lambda_{least}}{\lambda_{major}}}$ |
| Centroid Distance | $\left\Vert\vec{CoM}_{geom}-\vec{CoM}_{gl} \right\Vert_{2}$  where ${CoM}_{geom}$ is the geometric centroid and ${CoM}_{gl}$ is the centroid weighing each voxel by its corresponding intensity value |
| Approximate enclosing ellipsoid (AEE) based volume density | $\frac{3V}{4\pi abc}$  where $a, b, c$are the lengths of the ellipsoid’s semi principal axes obtained from PCA |
| AEE based area density | $\frac{A}{4\pi\left( \frac{a^{p}b^{p}+a^{p}c^{p}+b^{p}c^{p}}{3} \right)^{\frac{1}{p}}}$  where $p$ is a constant |
| Axis aligned bounding box (AABB) based volume density | $\frac{V}{lwh}$  where $l,w, h$ are length, width and height of the AABB |
| AABB based area density | $\frac{A}{2lw+2lh+2wh}$ |
| OMBB based volume density | $\frac{V}{qrs}$  where $q,r, s$ are length, width and height of the OMBB |
| OMBB based area density | $\frac{A}{2qr+2qs+2rs}$ |
| Minimum volume enclosing ellipsoid (MVEE) based volume density | $\frac{3V}{4\pi cde}$  where $c, d, e$are the lengths of the ellipsoid’s semi principal axes obtained from Khachiyan algorithm |
| MVEE based area density | $\frac{A}{4\pi\left( \frac{c^{p}d^{p}+c^{p}e+d^{p}e^{p}}{3} \right)^{\frac{1}{p}}}$ |
| Integrated intensity | $VI_{avg}$  where $I_{avg}$ is the average intensity of ROI |

**Supplementary Data**

Implementation of cross validation: <https://figshare.com/articles/dataset/Implementation_of_cross_validation_docx/23750700>


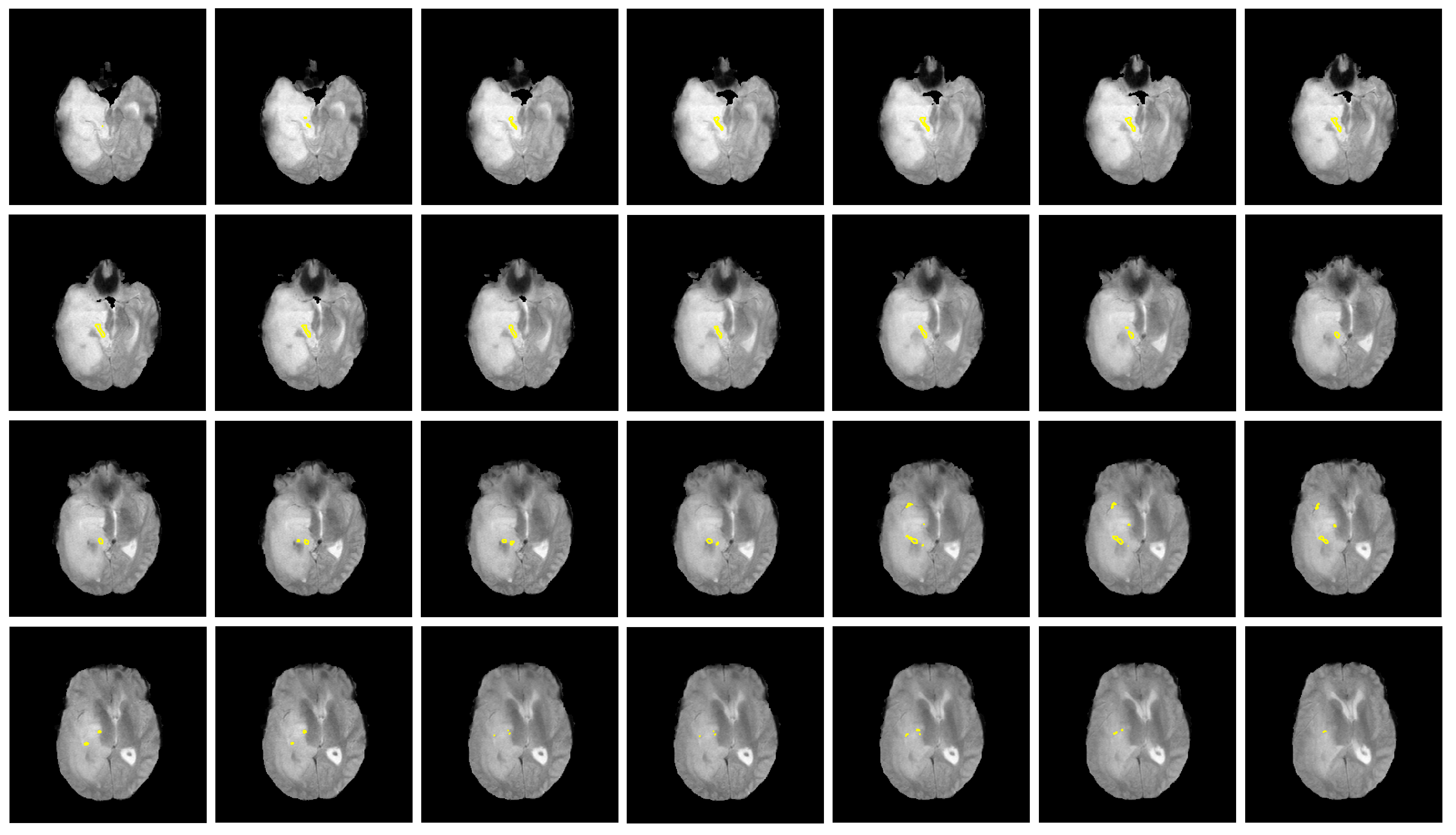


**Supplementary Figure 1.** Contoured ROIs (yellow) showing astrocytoma (Grade III) on contrast enhanced T1-weighted MR images.

Adapted from [CBICA Image Processing Portal; https://ipp.cbica.upenn.edu/.  A web accessible platform for imaging analytics; Center for Biomedical Image Computing and Analytics, University of Pennsylvania.](https://ipp.cbica.upenn.edu/%22https:/www.cbica.upenn.edu/CBICA_IPP_citation.bib/%22)


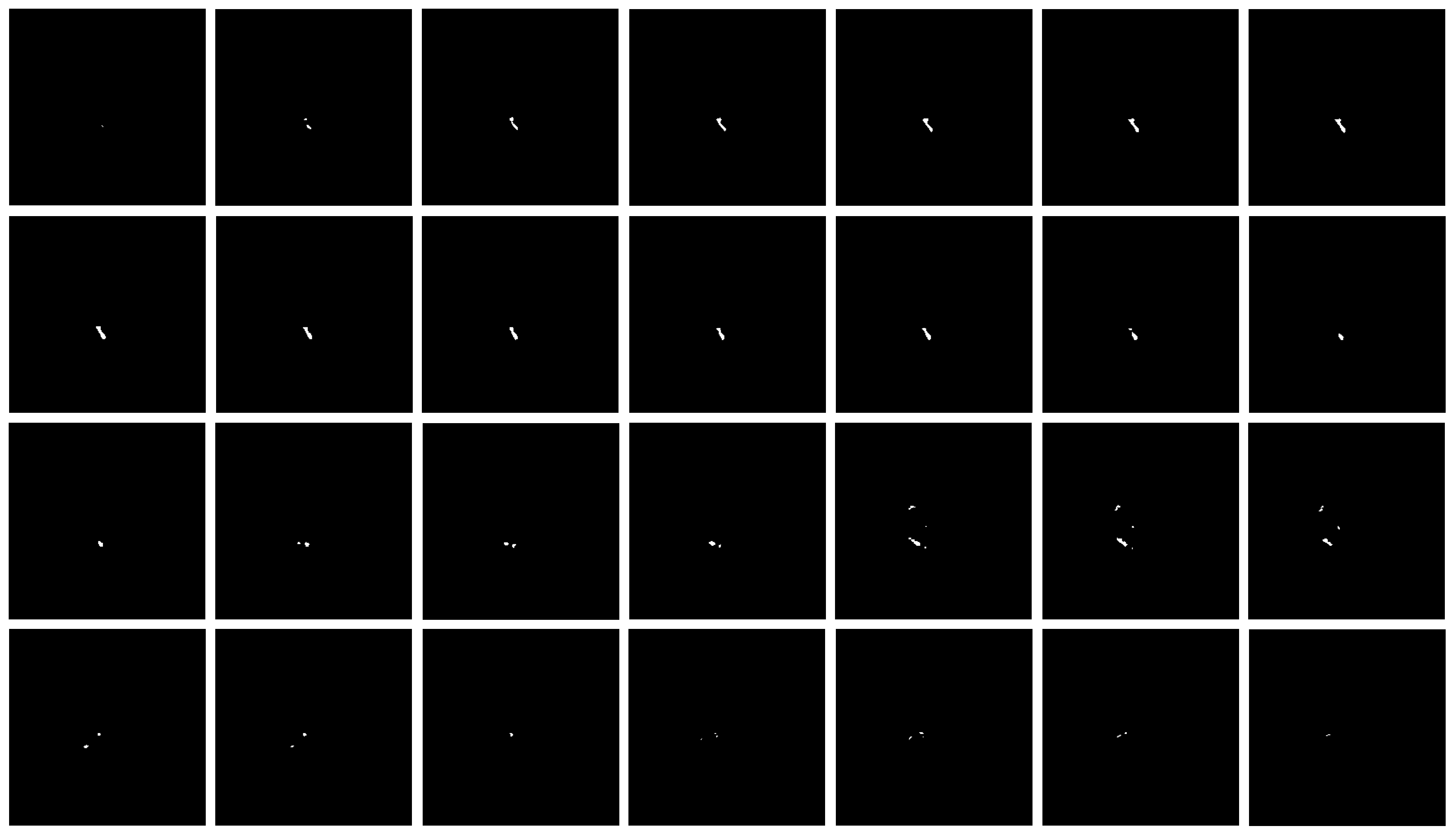


**Supplementary Figure 2.** Segmented ROIs corresponding to the contoured ROIs shown in Figure 1. Adapted from [CBICA Image Processing Portal; https://ipp.cbica.upenn.edu/.  A web accessible platform for imaging analytics; Center for Biomedical Image Computing and Analytics, University of Pennsylvania.](https://ipp.cbica.upenn.edu/%22https:/www.cbica.upenn.edu/CBICA_IPP_citation.bib/%22)


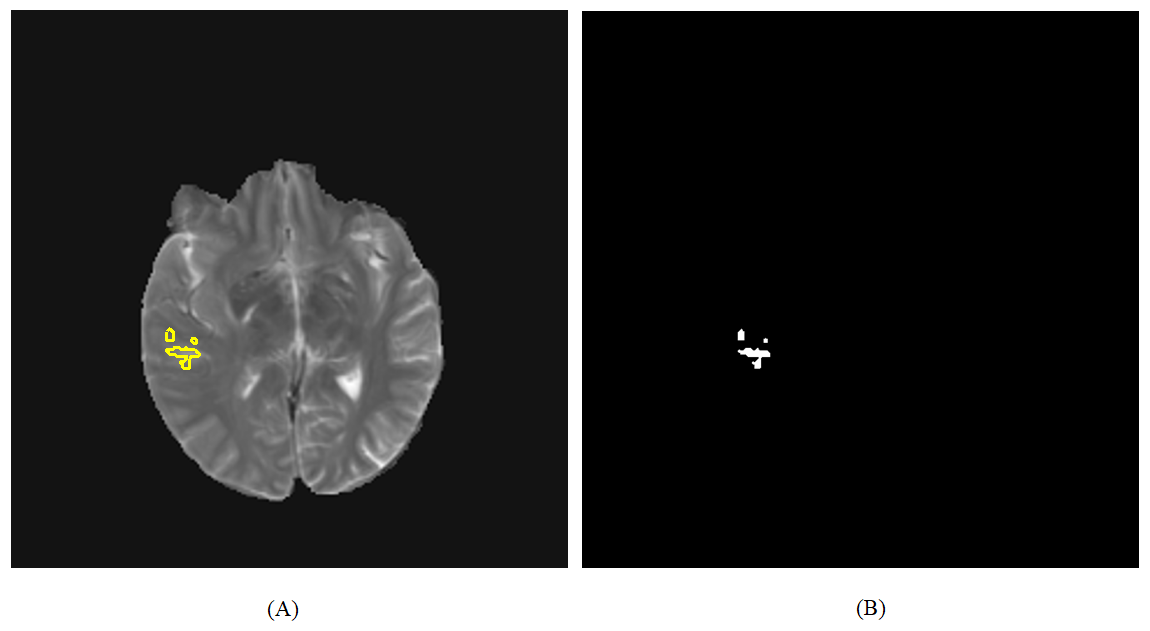


**Supplementary Figure 3.** (A) A contoured ROI (yellow) and (B) corresponding segmented ROI are shown (Magnification ×15; (A) relative to a slice in Supplementary Figure 1 and (B) relative to a slice in Supplementary Figure 2)


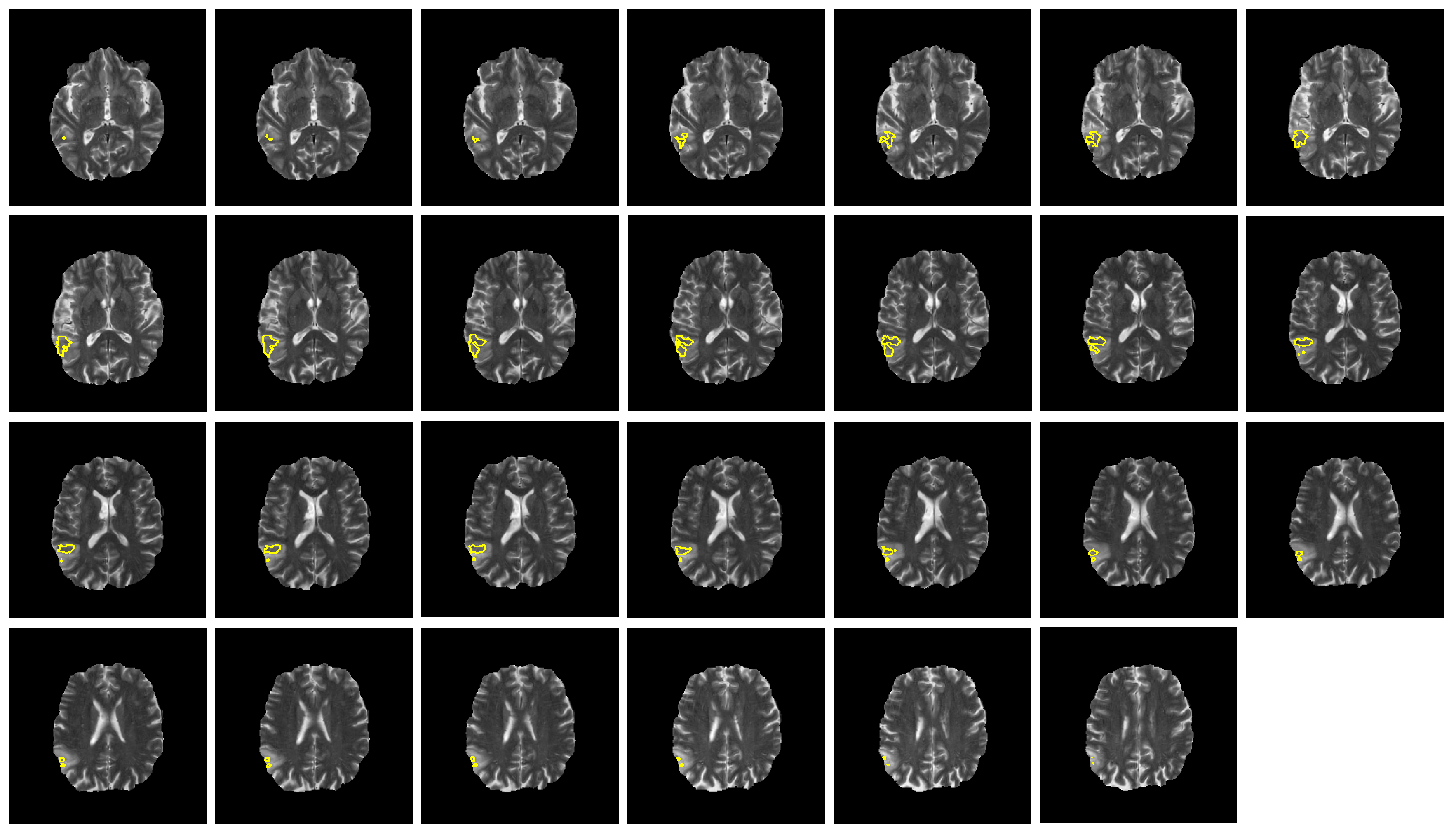


**Supplementary Figure 4.** Contoured ROIs (yellow) showing oligodendroglioma (Grade II) on contrast enhanced T1-weighted MR images.

Adapted from [CBICA Image Processing Portal; https://ipp.cbica.upenn.edu/.  A web accessible platform for imaging analytics; Center for Biomedical Image Computing and Analytics, University of Pennsylvania.](https://ipp.cbica.upenn.edu/%22https:/www.cbica.upenn.edu/CBICA_IPP_citation.bib/%22)


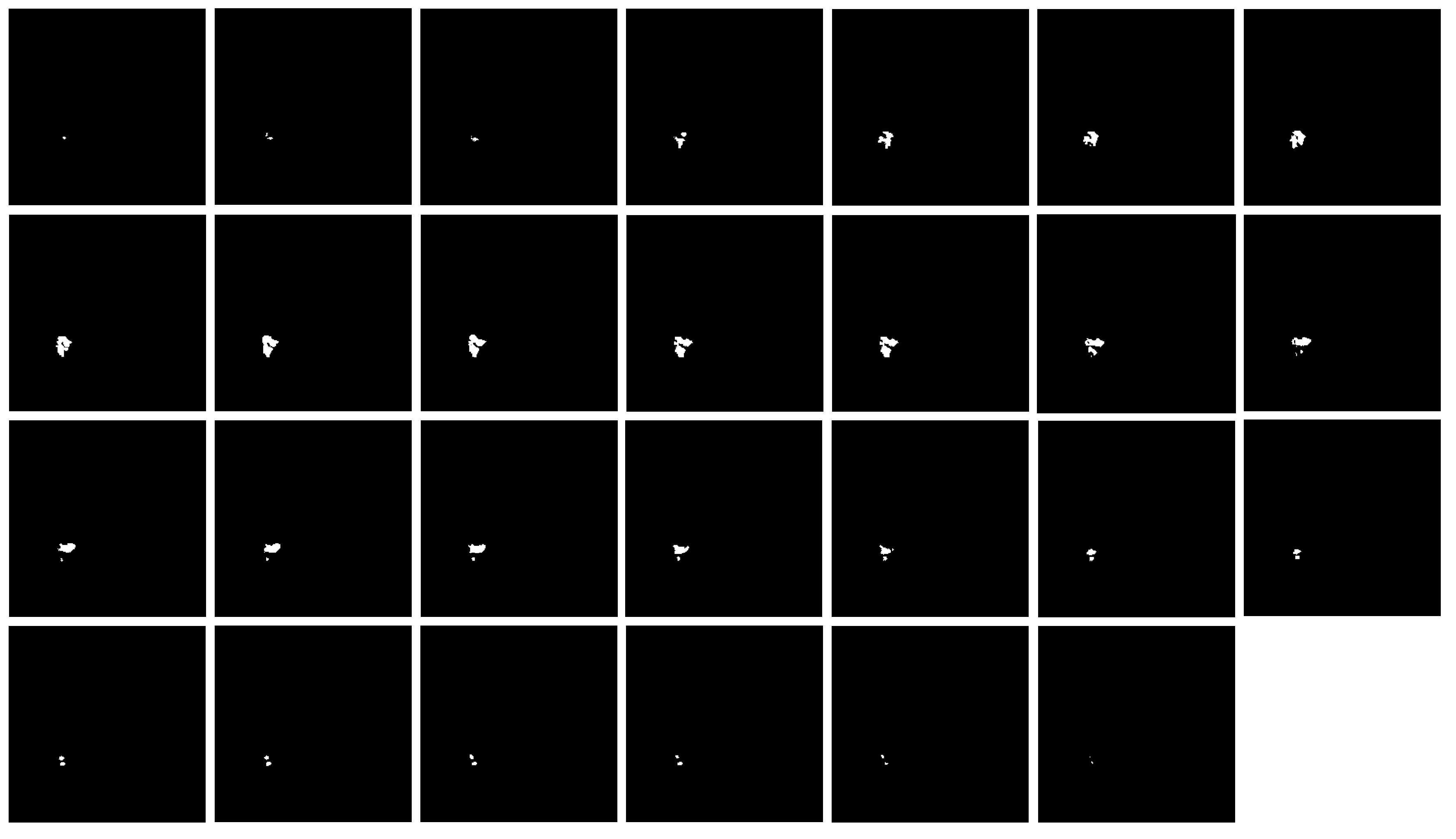


**Supplementary Figure 5.** Segmented ROIs corresponding to the contoured ROIs shown in Supplementary Figure 4.

Adapted from CBICA Image Processing Portal; https://ipp.cbica.upenn.edu/.  A web accessible platform for imaging analytics; Center for Biomedical Image Computing and Analytics, University of Pennsylvania.


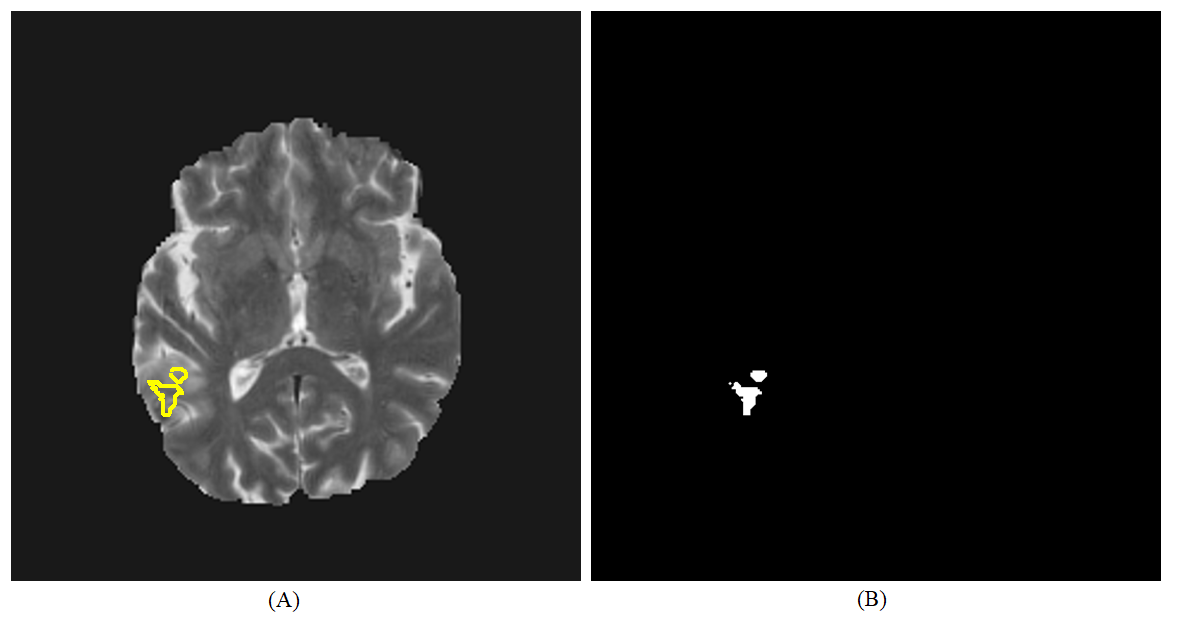


**Supplementary Figure 6.** (A) A contoured ROI (yellow) and (B) corresponding segmented ROI are shown (Magnification ×15; (A) relative to a slice in Supplementary Figure 1 and (B) relative to a slice in Supplementary Figure 2)
